# Effect of Decaffeinated Green Tea Polyphenols on Body Fat and Precocious Puberty in Obese Girls: A Randomized Controlled Trial

**DOI:** 10.3389/fendo.2021.736724

**Published:** 2021-10-12

**Authors:** Luyao Xie, Qingya Tang, Die Yao, Qiuyun Gu, Hao Zheng, Xiaodi Wang, Zhiping Yu, Xiuhua Shen

**Affiliations:** ^1^ Department of Clinical Nutrition, Xinhua Hospital Affiliated to Shanghai Jiao Tong University School of Medicine, Shanghai, China; ^2^ School of Public Health, Shanghai Jiao Tong University School of Medicine, Shanghai, China; ^3^ Department of Nutrition, Shanghai Jiao Tong University School of Medicine, Shanghai, China; ^4^ Department of Nutrition and Dietetics, University of North Florida, Jacksonville, FL, United States; ^5^ Shanghai Key Laboratory of Pediatric Gastroenterology and Nutrition, Xinhua Hospital Affiliated to Shanghai Jiao Tong University School of Medicine, Shanghai, China

**Keywords:** obesity, precocious puberty, decaffeinated green tea polyphenol, girls, randomized controlled trial

## Abstract

**Background:**

Obesity has been reported to be an important contributing factor for precocious puberty, especially in girls. The effect of green tea polyphenols on weight reduction in adult population has been shown, but few related studies have been conducted in children. This study was performed to examine the effectiveness and safety of decaffeinated green tea polyphenols (DGTP) on ameliorating obesity and early sexual development in girls with obesity.

**Design:**

This is a double-blinded randomized controlled trial. Girls with obesity aged 6–10 years old were randomly assigned to receive 400 mg/day DGTP or isodose placebo orally for 12 weeks. During this period, all participants received the same instruction on diet and exercise from trained dietitians. Anthropometric measurements, secondary sexual characteristics, B-scan ultrasonography of uterus, ovaries and breast tissues, and related biochemical parameters were examined and assessed pre- and post-treatment.

**Results:**

Between August 2018 and January 2020, 62 girls with obesity (DGTP group *n* = 31, control group *n* = 31) completed the intervention and were included in analysis. After the intervention, body mass index, waist circumference, and waist-to-hip ratio significantly decreased in both groups, but the percentage of body fat (PBF), serum uric acid (UA), and the volumes of ovaries decreased significantly only within the DGTP group. After controlling confounders, DGTP showed a significantly decreased effect on the change of PBF (*β* = 2.932, 95% CI: 0.214 to 5.650), serum UA (*β* = 52.601, 95% CI: 2.520 to 102.681), and ovarian volumes (right: *β* = 1.881, 95% CI: 0.062 to 3.699, left: *β* = 0.971, 95% CI: 0.019 to 1.923) in girls with obesity. No side effect was reported in both groups during the whole period.

**Conclusion:**

DGTP have shown beneficial effects of ameliorated obesity and postponed early sexual development in girls with obesity without any adverse effects.

**Clinical Trial Registration:**

[https://clinicaltrials.gov/ct2/show/NCT03628937], identifier [NCT03628937].

## Introduction

Precocious puberty refers to the appearance of physical and hormonal signs of pubertal development at an age earlier than normal. Female precocious puberty is defined as the development of secondary sexual characteristics before the age of 8 or menarche before the age of 10 (1). Currently, increasing numbers of countries worldwide are experiencing earlier sexual development in children (2–4). In China, a multi-center study in 2013 reported that the prevalence of precocious puberty in children was 0.43%, and the proportion of girls with breast development before the age of 8 was 2.91% (5). Also, a survey in nine major cities in China of about 20,654 girls found that 19.57% of urban girls aged 8 already had breast development (6). Moreover, the prevalence of precocious puberty is higher in girls, 5 to 10 times more common than in boys (7). Early sexual development in children was reported to be associated with rapid bone maturation with decreased adult height, psychological problems, and also increased risk of reproductive tract cancers in adulthoods (8).

The mechanisms of precocious puberty are complicated, which involve genetic, nutritional, psychosocial stress, and environmental toxin interactions (9, 10). Some studies demonstrated that obesity was an important contributing factor to early sexual development, especially for girls (11–16). According to the Third National Health and Nutrition Survey in the United States, Rosenfield et al. (17) reported that girls with high body mass index (BMI) had a higher proportion of breast development and pubic hair development, and the average age of menarche was 10.2 years old, 10 months earlier than the girls in the normal BMI range. Also, Biro’s study (18) suggested that the BMI of girls with precocious puberty was higher than that of normal development girls.

Gonadotropin-releasing hormone agonist therapy is well established as the standard treatment for central precocious puberty worldwide. In addition, some other clinical treatments like traditional Chinese medicine and acupuncture therapy were also used. Though clinically precocious puberty can be treated in some ways, it is important to find approaches to prevent or delay its development in children.

Green tea, originated in China, is made from the leaves of *Camellia sinensis* and contains high quantities of polyphenols (19). Among the large body of polyphenols, epigallocatechin gallate (EGCG) has been shown effective in reducing body weight, along with decreased adipocyte differentiation and proliferation during lipogenesis (20). A high dose of catechins ingestion (~600 mg/day), has also been reported to lower body fat and cholesterol in adults with obesity (21). Till now, most human studies on green tea polyphenols (GTP) were conducted in adult populations, and only a Japanese study conducted in children (22), which showed that 576 mg catechins for 24 weeks improved obesity and suppressed leptin concentration without raising any safety concerns for children with obesity. It is worth mentioning that the caffeine contained in tea polyphenols might cause main adverse effects for children, such as sleep difficulty (23), mental anxiety and depression (24), and slow heart rate (25). It is probably for this reason that GTP interventions were rarely conducted in children with obesity. Decaffeinated green tea polyphenols (DGTP) thus become more appealing to be tested for their weight reduction effect in children.

Since precocious puberty is a common comorbidity in girls with obesity (26), it is important to find effective ways to manage obesity as well as to prevent precocious puberty. Previously, our group has conducted a pilot experiment in female Sprague Dawley (SD) rats, and reported that GTP could delay the date of vaginal opening of precocious puberty rats (*p* < 0.05) (27), showing the preliminary effect of GTP. To our best knowledge, the potential effects of GTP or DGTP to delay precocious puberty has not yet been tested in children with obesity. Therefore, this study aimed to examine the effect of DGTP on obesity and sexual development in girls with obesity aged 6–10 years old, and the safety of the study was also assessed.

## Materials and Methods

### Study Design and Participants

We did a randomized, double-blinded, placebo-controlled trial that enrolled obese girls with a 12-week DGTP or placebo intervention period. This study was registered with a Clinical Trials.gov identifier NCT03628937 and was approved by the Ethics Committee of Xinhua Hospital (Shanghai, China). Participants were recruited from the Clinical Nutrition Department, Children Endocrinology Department, Children Health Care Department in Xinhua Hospital, and some primary schools near the hospital from August 2018 to January 2020. Flyers and posters with the study information were posted in the hospital, primary school campus, and also posted on the hospital and external websites (such as WeChat, which is one of the most common online social platforms in China). The inclusion criteria for this study were as follows: (1) girls with obesity aged 6–10 years old; (2) BMI reached or exceeded the overweight threshold of school-aged girls in China (28) (6 years old, BMI ≥16.3 kg/m^2^; 7 years old, BMI ≥17.2 kg/m^2^; 8 years old, BMI ≥18.1 kg/m^2^; 9 years old, BMI ≥19.0 kg/m^2^; 10 years old, BMI ≥20.0 kg/m^2^). Participants with the following conditions were excluded: (1) menarche or central precocious puberty clearly diagnosed; (2) current treatment for obesity; (3) taking medications that would impact obesity, such as the stimulants and some psychotropic drugs; (4) severe systemic, liver, and renal disorders; and (5) judged to be ineligible by the study physician, such as mental disorders.

During recruitment, the study details and possible risks of the study were explained to participants and their guardians both in written form and orally by the investigators. Written informed consent for publication of their clinical details was obtained from each participant or her guardian.

### Randomization and Masking

After recruitment, girls with obesity were randomly assigned (on a 1:1 ratio) to the DGTP group or the control group by computer-generated block randomization with block sizes of 4, conducted by the Clinical Research Unit of Shanghai Xinhua Hospital, China. The random results were sealed and given to a staff not involved in the trial investigation. For each new participant, the staff informed doctors the group of the participant orderly. The participants, guardians, outcome assessors, statisticians, conclusion drawers, as well as all doctors or dietitians were masked to the treatment allocation, except for the investigator who contacted DGTP capsule manufacturing companies and distributed the DGTP and placebo capsules to participants. Both DGTP and placebo were packed in the congruent opaque capsules for avoiding light and blinding. The two types of study capsules were assigned to transparent bottles of exactly the identical size and appearance, except for different label numbers on the bottles to distinguish their types. The randomization list and questionnaires were securely stored in a locker, and the data were stored in the researcher’s computer protected by a password.

### Procedures

The DGTP were purchased from CHENGDU WAGOTT BIO-TECH CO., LTD (Chengdu City, China), and the capsules, including the placebo capsules, were manufactured by LIAOCHENG AOJIAN BIO-TECH CO., LTD (Shandong Province, China). The capsules were made from food additive gelatin with soybean oil and DGTP as filler, and all materials had certificates of safety testing and analysis.

After the informed consent and randomization, participants in the DGTP group were asked to consume two capsules daily (200 mg/capsule DGTP, EGCG accounted for 50%, caffeine accounted for lower than 0.5%), while the control group was given indistinguishable capsules containing isodose soybean oil as a placebo treatment. The dosage of 400 mg daily DGTP (EGCG accounted for 50%) was verified to be safe for children in a previous study (22). The intervention period would last for 12 weeks, which was consistent with some previous GTP interventions among adults with weight reduction effectively and no adverse effect (21, 29–32). Each participant took two capsules 30 min post-dinner per day for 12 weeks. During the intervention period, besides DGTP or placebo treatments, participants were instructed to consume an almost isocaloric diet and engage in the same level of physical activities every day. Participants were also reminded not to receive other types of obesity management, or consume catechins, or polyphenol-rich foods and beverages during the study period. The participants’ guardians were responsible for providing their children with the recommended diets, and study investigators contacted the guardians regularly to ensure the compliance of participants and also monitor the potential adverse effects of treatment.

At baseline, participants were asked to complete demographic and lifestyle questionnaires, and bone age measurement. Other assessments were conducted at baseline and at the end of the 12-week intervention period, mainly including anthropometric measurements; body composition analysis; secondary sexual characteristics; B ultrasonic measurements in uterus, ovaries, and breast tissues; biochemical parameter measurements; and dietary assessments. The safety assessments were conducted during the whole study period. A timeline of the study is shown in **Table 1**.

**Table 1:** Study design and timeline^1^.

Period	Screening	Baseline	Intervention			Follow-up	End
Week (W)	-1W	0W	1W	6W	12W	W13 -	Menarche
***Screening and enrollment***							
Eligibility	⚪						
Informed consent	⚪						
Confirm suitability for study	⚪						
Randomization		⚪					
Demography and lifestyle questionnaires		⚪					
Food frequency questionnaire		⚪					⚪
***Intervention***							
DGTP intake (Experimental Group)			400 mg/d				
Placebo intake (Control Group)			400 mg/d				
***Assessments***							
Anthropometric measurements		⚪		⚪	⚪		⚪
Body composition analysis		⚪		⚪	⚪		⚪
Secondary sexual characteristics		⚪		⚪	⚪		⚪
Bone age		⚪					⚪
B ultrasonic examination in uterus, ovarian and breast		⚪			⚪		⚪
Biochemical parameters measurements		⚪			⚪		⚪
Diet and exercise diary			⚪	⚪	⚪		
DGTP capsule diary			⚪	⚪	⚪		
24-hour diet recall questionnaire							
WeChat or telephone access	⚪					⚪	
Self-report the onset of menarche							⚪
Safety of DGTP capsule			⚪	⚪	⚪		
Discomfort and acceptance of DGTP capsule			⚪		⚪		
Adverse event			⚪		⚪	⚪	⚪
Compliance			⚪		⚪	⚪	⚪

^1^DGTP, decaffeinated green tea polyphenols.

### Outcomes

The primary outcome was the effect of 12-week DGTP on percentage of body fat (PBF) change. The PBF of participants was measured using bioelectrical impedance analyses by whole-body impedance (InBody720, Biospace Inc., Korea) pre- and post-treatment. The onset of menarche was reported to be closely related with the achievement of a certain PBF in girls. Secondary efficacy outcomes included the changes from baseline in BMI, fat-free mass (FFM), serum triglyceride, total cholesterol, and uric acid (UA); secondary sexual characteristics; volume of uterus, ovary, and breast; and sex hormones levels at week 12. The blood samples were collected in the morning with the participants in fasting state, and the serum was sent for laboratory test. Serum triglyceride, total cholesterol, and uric acid values were detected by uricase method using a Hitachi 7600 automatic biochemical analyzer (Hitachi, Tokyo, Japan), and sex hormone levels were measured with a chemiluminescence immuno-metric assay using a UniCell DXI 800 analyzer (Beckman Coulter Inc., Fullerton, CA, USA). Also, the average age of onset of menarche will be followed up and recorded in participants as a secondary outcome.

Participants and their guardians were asked to record the number of capsules participants took every day and all adverse events occurred during the study. Side effects and adverse reactions on sleep or digestive tract were assessed at baseline, week 1, week 6, week 12, and 3 months follow-up after the intervention. Meanwhile, the serum trace elements, hepatic and renal function, and complete blood count of participants were also measured at baseline and week 12 to ensure safety. All participants were free to withdraw at any time during the study.

### Statistical Analysis

Data were analyzed using SPSS version 22.0 software (SPSS Inc., Chicago, IL, USA). Continuous variables are presented as the mean ± SD, and categorical variables are presented as number and percentages. To compare differences between the DGTP group and the control group, independent sample *t*-test was performed for continuous variables and *χ* ^2^ test for categorical variables. To compare differences between baseline and after 12-week intervention in the same groups, paired *t*-test was performed for continuous variables, and McNemar test was performed for matched categorical variables. In addition, the group differences in changes from week 0 to week 12 were calculated and analyzed using independent sample *t*-test and *χ* ^2^ test as well.

Multiple linear regression was employed to evaluate the effect of DGTP intake on the changes of obese and pubertal related outcomes after controlling confounding factors in girls with obesity. All *p* values were calculated based on two-sided test, and *p* < 0.05 was considered statistically significant.

## Results

**Figure 1:**
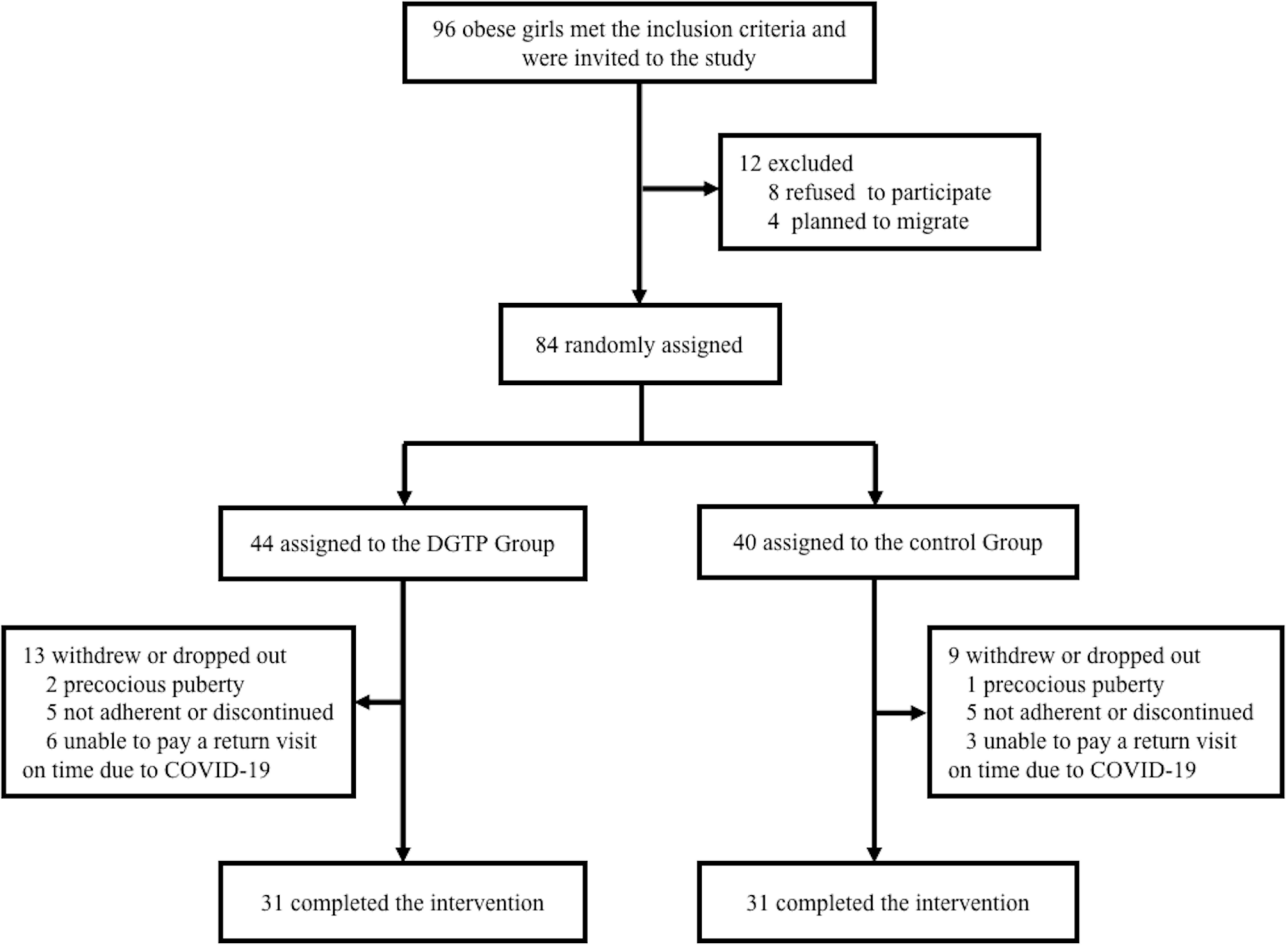
Participant flow diagram showing obese girls aged 6–10 years old from screening to endline analysis. Boxes display number of participants included in the analysis in each stage, along with the reasons for loss of follow-up or exit. COVID-19, Coronavirus Disease 2019; DGTP, decaffeinated green tea polyphenols.

After screening, 96 girls with obesity who met the inclusion criteria were invited to participate. Among them, eight girls refused to participate, while four others were excluded due to plans of moving out of town. The remaining 84 participants were enrolled and were randomly allocated into either the DGTP group or the placebo control group. During the study, 13 participants in the DGTP group and 9 in the control group withdrew or dropped out, and the dropout rate was 22/84 (26.2%). Specifically, in the 22 dropouts, 9 could not pay a punctual study visit due to the impact of the coronavirus disease 2019 (COVID-19) in early 2020, which was the period of the initial outbreak of this pandemic, and the original arrangements of follow-up in this study were unavoidably affected. Also, two girls in the DGTP group and one in the control group were diagnosed as precocious puberty and changed to gonadotropin-releasing hormone agonist therapy. Five participants in the DGTP group and five in the control group were considered non-adherent or discontinued the study due to personal reasons like too overload homework to cooperate with. Finally, 62 participants completed the study without any adverse events or side effects reported. The study flowchart is in **Figure 1**.

**Table 2** presents the demographic and lifestyle data of participants at baseline. There were no significant differences between groups in almost all measures except that a higher percentage of DGTP participants had screen time over 2 h a day than placebo participants (36% *vs*. 10%; *p* < 0.05).

**Table 2:** Demographic characteristics and lifestyles in both DGTP group and control group at baseline^1^.

	DGTP group (*n* = 31)	Control group (*n* = 31)
Age, years	8.1 (1.3)	8.4 (1.2)
Birth place		
Shanghai City	26 (84)	28 (90)
Other cities in China	5 (16)	3 (10)
Birth weight, kg	3.4 (1.1)	3.6 (0.6)
Birth body length, cm	49.9 (0.8)	50.1 (2.1)
Age of obesity onset, years	3.7 (2.8)	3.8 (2.5)
Picky eater	11 (36)	8 (26)
Having breakfast per day	26 (84)	29 (94)
Past eating habits		
More meats and less vegetables	14 (45)	9 (29)
More vegetables and less meats	14 (45)	18 (58)
Balance of meats and vegetables	2 (7)	4 (13)
Weekly physical activity, h	6.2 (2.5)	5.4 (2.3)
Screen time^*^		
<1 h/day	12 (39)	15 (48)
1–2 h/day	7 (23)	13 (42)
>2 h/day	11 (36)	3 (10)
Sleep time		
<8 h/day	3 (10)	1 (3)
8–10 h/day	27 (87)	29 (94)
>10 h/day	0	1 (3)
Father’s education level		
Middle school or lower High school or vocational college	2 (7) 13 (42)	4 (13) 9 (29)
Undergraduate or higher	15 (48)	18 (58)
Mother’s education level		
Middle school or lower	2 (7)	4 (13)
High school or vocational college	8 (26)	10 (32)
Undergraduate or higher	20 (65)	17 (55)
Household income		
<♆8,000/month	2 (7)	6 (19)
♆8,000–15,000/month	9 (29)	7 (23)
♆15,000–50,000/month	15 (48)	18 (58)
>♆50,000/month	4 (13)	0
Mother’s age of menarche, years	13.1 (1.1)	12.8 (1.4)
Father’s age of first spermatorrhea, years	14.2 (1.2)	14.4 (0.7)
Mother’s age of pregnancy, years	28.5 (3.6)	28.5 (2.9)

^1^Data are expressed as n (%) for categorical variables or mean (SD) for continuous variables. ^*^Statistical significance when comparing DGTP and control groups at baseline. DGTP, decaffeinated green tea polyphenols.

Anthropometric and body composition data are presented in **Table 3**. Regarding anthropometric data, there was no significant difference between the two groups at baseline and after the intervention. When comparing pre- and post-intervention differences within each group, the height of both groups significantly increased (DGTP group: Δheight: 2.3 ± 0.9 cm, Control group: Δheight: 2.4 ± 0.9 cm), and the BMI, waist circumference (WC), waist-to-hip ratio (WHR), and waist-to-height ratio (WHtR) of both groups significantly decreased after 12-week intervention (DGTP group: ΔBMI: −0.8 ± 1.4 kg/m^2^, Control group: ΔBMI: −0.7 ± 1.4 kg/m^2^; DGTP group: ΔWC: −2.8 ± 5.4 cm, Control group: ΔWC: −2.5 ± 4.9 cm; DGTP group: ΔWHR: −0.03 ± 0.05, Control group: ΔWHR: −0.03 ± 0.06; DGTP group: ΔWHtR: −0.03 ± 0.04, Control group: ΔWHtR: −0.03 ± 0.04; *p* < 0.05). No significant changes were found in weight, hip circumference, and blood pressure in either group (**Table 3**). As for within-group analysis, in the DGTP group, the basal metabolic rate and FFM significantly increased (Δbasal metabolic rate: 18.0 ± 39.9 kcal; ΔFFM: 0.7 ± 1.6kg) while the fat mass and PBF significantly decreased after the 12-week intervention (Δfat mass: 0.7 ± 2.4 kg; ΔPBF: −1.8 ± 4.3%).

**Table 3:** Changes in anthropometrics and body composition after daily DGTP intervention for 12 weeks^1^.

	DGTP group (*n* = 31)			Control group (*n* = 31)		
	Baseline	After intervention	Change at week 12	Baseline	After intervention	Change at week 12
*Anthropometrics*						
Height, cm	136.6 (7.5)	138.8 (7.6)^†^	2.3 (0.9)	139.3 (8.4)	141.7 (8.2)^‡^	2.4 (0.9)
Weight, kg	42.5 (7.5)	43.7 (9.4)	1.2 (7.0)	46.0 (10.3)	46.3 (9.9)	0.3 (1.7)
BMI, kg/m^2^	22.6 (2.9)	21.9 (2.7)^†^	−0.8 (1.4)	23.5 (3.3)	22.8 (2.9)^‡^	−0.7 (1.4)
WC, cm	76.2 (7.4)	73.4 (8.2)^†^	−2.8 (5.4)	78.4 (8.6)	75.9 (7.5)^‡^	−2.5 (4.9)
HC, cm	83.4 (6.1)	83.3 (5.6)	−0.2 (4.8)	85.8 (7.6)	86.3 (7.6)	0.5 (3.5)
WHR	0.91 (0.06)	0.88 (0.06)^†^	−0.03 (0.05)	0.91 (0.05)	0.88 (0.06)^‡^	−0.03 (0.06)
WHtR	0.56 (0.04)	0.53 (0.05)^†^	−0.03 (0.04)	0.56 (0.05)	0.54 (0.04)^‡^	−0.03 (0.04)
Systolic blood pressure, mmHg	106.1 (12.7)	106.1 (11.0)	0.00 (11.7)	105.8 (13.8)	107.0 (16.0)	1.2 (15.8)
Diastolic blood pressure, mmHg	72.2 (20.3)	71 (12.4)	−1.2 (21.2)	66.8 (10.8)	70.2 (14.1)	3.4 (13.3)
*Body composition*						
Fat mass, kg	15.6 (4.6)	14.9 (4.5)^†^	−0.7 (2.4)	16.17 (5.0)	16.4 (5.5)	0.3 (4.2)
Skeletal muscles, kg	13.9 (2.5)	14.3 (2.6)	0.4 (1.0)	15.4 (2.8)^*^	15.8 (3.2)	0.2 (1.7)
FFM, kg	27.0 (4.1)	27.7 (4.4)^†^	0.7 (1.6)	29.3 (4.9)	29.9 (5.6)	0.6 (2.3)
PBF, %	36.0 (5.3)	34.2 (4.9)^†^	−1.8 (4.3)	35.0 (6.1)	35.1 (5.4)	0.1 (5.0)
Basal metabolic rate, kcal	950.3 (91.2)	968.3 (94.1)^†^	18.0 (39.9)	1,008.3 (104.4)^*^	1,013.3 (122.5)^‡^	5.0 (62.0)

^1^Data are expressed as mean (SD) for all continuous variables. ^*^Statistical significance when comparing DGTP and control groups at baseline. ^†^Change statistical significance after 12 weeks within the DGTP group. ^‡^Change statistical significance after 12 weeks within the control group. BMI, body mass index; DGTP, decaffeinated green tea polyphenols; FFM, fat-free mass; HC, hip circumference; PBF, percentage of body fat; WC, waist circumference; WHR, waist-to-hip ratio; WHtR, waist-to-height ratio.

As shown in **Table 4**, at baseline, the bone age of participants in the DGTP group and the control group were 9.6 ± 1.5 years and 9.7 ± 1.6 years, both higher than their actual age. The difference between bone age and actual age in the two groups was 1.56 ± 1.43 years and 1.48 ± 0.93 years, respectively, with no statistical difference between groups. For the secondary sexual characteristics and ultrasonic examination results, no significant group differences were found either at baseline or after the intervention. When comparing pre- and post-intervention changes, the number of participants with breast Tanner stage II or above and the uterine volume in both groups significantly increased after 12 weeks (DGTP group: Δuterine volume: 0.30 ± 2.20 cm^3^, Control group: Δuterine volume: 0.46 ± 1.80 cm^3^). In addition, in the DGTP group, ovarian volumes on both sides significantly decreased (Right: 0.24 ± 1.47 cm^3^; Left: 0.39 ± 1.24 cm^3^) after 12 weeks, and the number of participants with diameter >4 mm follicles in the left ovary reduced by three (*p* < 0.05).

**Table 4:** Changes in secondary sexual characteristics and ultrasonic examination in breast, uterus, and ovary tissues after daily DGTP intervention for 12 weeks^1^.

	DGTP group (*n* = 31)			Control group (*n* = 31)		
	Baseline	After intervention	Change at week 12	Baseline	After intervention	Change at week 12
Age, years	8.1 (1.3)	–	–	8.4 (1.2)	–	–
Bone age, years	9.6 (1.5)	–	–	9.7 (1.6)	–	–
ΔBone age − age, years	1.56 (1.43)	–	–	1.48 (0.93)	–	–
*Secondary sexual characteristics*						
Having pubic hairs	0	1 (3)	+1	4 (13)	5 (16)	+1
Having armpit hairs	2 (7)	3 (10)	+1	3 (10)	7 (23)	+4
Breast Tanner stage II or above	9 (29)	17 (55)^†^	+8	13 (42)	16 (52)^‡^	+3
*B ultrasonic measurements in breast, uterus and ovary tissues*						
Appearing breast tissue	19 (61)	19 (61)	0	16 (52)	16 (52)	0
Area of right breast tissue, cm^2^	3.1 (3.6)	2.4 (2.3)	−0.7 (2.7)	4.6 (10.8)	2.9 (2.7)	−1.7 (8.0)
Area of left breast tissue, cm^2^	2.9 (3.2)	2.9 (3.2)	−0.4 (298.3)	4.8 (9.2)	2.8 (2.5)	−1.5 (7.1)
Uterine volume, cm^3^	2.13 (1.57)	2.42 (2.40)^†^	0.30 (2.20)	2.14 (1.74)	2.60 (2.41)^‡^	0.46 (1.80)
Right ovarian volume, mm^3^	2.30 (1.65)	2.06 (1.38)^†^	−0.24 (1.47)	2.04 (1.36)	2.64 (2.43)	0.60 (2.70)
Left ovarian volume, mm^3^	2.20 (1.43)	1.82 (1.37)^†^	−0.39 (1.24)	1.82 (1.14)	2.20 (1.65)	0.39 (1.77)
With diameter >4 mm follicles in right ovary	6 (19)	6 (19)	0	7 (23)	6 (19)	−1
With diameter >4 mm follicles in left ovary	6 (19)	3 (10)^†^	−3	7 (23)	6 (19)	−1

^1^Data are expressed as n (%) for categorical variables or mean (SD) for continuous variables. ^†^Change statistical significance after 12 weeks within the DGTP group. ^‡^Change statistical significance after 12 weeks within the control group. DGTP, decaffeinated green tea polyphenols.

As shown in **Table 5**, no significant group differences in biomarkers of obesity or sexual development were found at baseline. After 12 weeks, the serum UA concentration in the DGTP group had a significantly higher drop than the control group. When examining the pre- and post-intervention changes within each group, in the DGTP group the serum UA concentration significantly dropped from 376.42 ± 91.08 to 326.65 ± 53.99 μmol/L after 12 weeks. In addition, the concentrations of sex hormones including estradiol, follicle-stimulating hormone, luteinizing hormone in the DGTP group, and estradiol, luteinizing hormone, and testosterone in the control group significantly increased after 12 weeks. However, those concentrations were still relatively low, far below the concentrations of sexual development initiation of girls.

**Table 5:** Changes in the levels of serum sex hormones, lipid profiles, and uric acid after daily DGTP or placebo intervention for 12 weeks^1^.

	DGTP group (*n* = 31)			Control group (*n* = 31)		
	Baseline	After intervention	Change at week 12	Baseline	After intervention	Change at week 12
Total cholesterol, mmol/L	4.36 (0.73)	4.15 (0.48)	−0.13 (0.58)	4.40 (0.97)	4.37 (0.86)	−0.027 (0.99)
Triglyceride, mmol/L	1.15 (0.67)	0.80 (0.57)	−0.35 (0.58)	1.32 (0.67)	0.61 (0.28)	−0.71 (0.62)
Uric acid, μmol/L	376.42 (91.08)	326.65 (53.99)^†^	−47.97 (83.17)	336.37 (61.12)	336.35 (68.91)	−0.01 (69.06)^§^
E2, pmol/L	52.37 (34.49)	73.59 (45.97)^†^	21.21 (52.55)	53.79 (42.81)	76.61 (51.41)^‡^	22.82 (44.04)
FSH, IU/L	2.81 (1.85)	3.51 (2.30)^†^	0.70 (1.50)	3.28 (2.15)	3.53 (2.14)	0.25 (1.35)
LH, IU/L	0.53 (0.74)	0.90 (1.29)^†^	0.36 (0.79)	0.63 (1.02)	0.81 (1.21)^‡^	0.18 (0.46)
PRL(1), mIU/L	202.8 (147.1)	166.79 (105.37)	−50.79 (142.79)	170.48 (69.83)	165.81 (47.05)	−4.67 (68.28)
Testosterone, nmol/L	0.51 (0.48)	0.66 (0.50)	0.15 (0.39)	0.47 (0.49)	0.70 (0.56)^‡^	0.23 (0.39)

^1^Data are expressed as mean (SD) for all continuous variables. ^†^Change statistical significance after 12 weeks within the DGTP group. ^‡^Change statistical significance after 12 weeks within the control group. ^§^Significant difference when comparing DGTP and control groups in the Δvalue at Week 12. DGTP, decaffeinated green tea polyphenols; E2, estradiol; FSH, follicle-stimulating hormone; LH, luteinizing hormone; PRL(1), prolactin(1); TC, serum total cholesterol; TG, serum triglyceride; UA, serum uric acid.

The nutrient intake data are presented in **Table 6**. No statistical difference in nutrient intakes between the two groups was found before or after intervention except for the energy ratio of protein. After 12 weeks, the energy ratio of lipid decreased, and the energy ratio of carbohydrates increased in both groups. This might be due to the dietary instruction on limiting the excess consumption of lipids, sugars, and beverages during the study. With those changes, the proportion of three macronutrients seemed more balanced compared to the baseline.

**Table 6:** The nutrients intakes in two groups at baseline and after 12-week intervention^1^.

	DGTP group (*n* = 31)			Control group (*n* = 31)		
	Baseline	After intervention	Change at week 12	Baseline	After intervention	Change at week 12
Energy, kcal/day	1,384.5 (540.9)	1,332.8 (276.7)	−51.7 (610.1)	1,378.7 (464.8)	1,219.9 (387.3)	−158.8 (671.0)
Protein, g/day	56.3 (23.8)	48.4 (13.9)	−8.0 (25.8)	48.1 (16.7)	28.6 (16.8)	0.6 (21.7)
Lipid, g/day	66.2 (30.7)	74.2 (26.8)	212.4 (12.19)	72.9 (26.8)	50.2 (17.0)^‡^	−22.7 (34.9)
Carbohydrate, g/day	141.0 (70.4)	163.1 (45.6)	22.1 (85.1)	132.8 (57.4)	156.4 (47.9)	23.6 (77.2)
Dietary fiber, g/day	5.6 (3.2)	8.06 (8.26)	2.46 (7.40)	5.4 (2.6)	4.94 (3.30)	−0.42 (3.81)
Cholesterol, mg/day	478.2 (204.9)	345.3 (212.1)^†^	−132.9 (281.4)	425.4 (228.5)	388.8 (262.7)	−36.6 (348.3)
Vitamin A, μgRE/day	657.3 (508.7)	512.3 (365.4)	−145.0 (478.3)	700.7 (388.8)	424.2 (204.3)^‡^	−276.6 (444.9)
Vitamin D, μg/day	4.1 (4.0)	2.04 (3.95)^†^	−2.01 (3.85)	3.33 (3.95)	2.01 (2.99)	−1.32 (5.39)
Vitamin E, mg/day	23.6 (6.0)	19.9 (6.3)^†^	−3.7 (7.8)	21.6 (3.0)	20.0 (6.8)	−1.6 (7.7)
Vitamin C, mg/day	81.7 (107.8)	76.8 (72.0)	−4.9 (69.3)	71.3 (41.8)	51.7 (39.9)^‡^	−19.5 (52.3)
Folate, μg/day	87.7 (141.6)	56.1 (53.3)	−31.6 (148.3)	110.4 (78.4)	38.6 (32.8)^‡^	−71.8 (81.6)
Calcium, mg/day	465.2 (278.2)	445.8 (208.2)	−19.4 (330.1)	455.2 (186.1)	439.2 (245.8)	−16.0 (284.8)
Iron, mg/day	11.8 (6.2)	13.4 (5.5)	1.5 (6.7)	11.7 (4.4)	12.4 (5.4)	0.6 (6.5)
Zinc, mg/day	9.58 (5.19)	7.54 (2.07)	−2.03 (5.43)	8.21 (3.03)	7.62 (3.11)	−0.59 (4.04)
Selenium, μg/day	36.3 (15.9)	37.0 (39.9)	0.8 (33.0)	35.7 (15.3)	43.9 (36.4)	8.2 (31.6)
Energy ratio of protein	16.6 (4.4)	14.5 (3.7)	−0.02 (0.06)	14.03 (2.76)^*^	15.52 (4.81)	0.02 (0.05)^§^
Energy ratio of lipid	43.2 (9.3)	36.5 (6.6)^†^	−0.07 (0.1)	47.6 (8.7)	35.8 (7.7)^‡^	−0.12 (0.12)
Energy ratio of carbohydrate	40.2 (9.4)	49.0 (7.7)^†^	0.09 (0.1)	38.7 (10.1)	48.6 (7.3)^‡^	0.1 (0.1)

^1^Data are expressed as mean (SD) for all continuous variables. ^*^Statistical significance when comparing DGTP and control groups at baseline. ^§^Significant difference when comparing DGTP and control groups in the Δvalue at Week 12. ^†^Change statistical significance after 12 weeks within the DGTP group. ^‡^Change statistical significance after 12 weeks within the control group. DGTP, decaffeinated green tea polyphenols.

After controlling various confounding factors, the association between DGTP intake and the changes of obesity- or sexual development-related parameters after intervention are presented in **Table 7**. In model 1, DGTP intake was significantly associated with decreased ΔUA (*β* = 48.747, 95% confidence interval CI: 7.100 to 90.394, *p* = 0.02) in girls with obesity. In model 2, DGTP intake remained significantly associated with decreased ΔUA (*β* = 55.172, 95% CI: 6.328 to 104.015, *p* = 0.03) and also associated with reduced Δright ovarian volume (*β* = 1.935, 95% CI: 0.336 to 3.533, *p* = 0.02). Furthermore, in model 3, DGTP intake for 12 weeks was significantly associated with ΔPBF (*β* = 2.932, 95% CI: 0.214 to 5.650, *p* = 0.04), ΔUA (*β* = 52.601, 95% CI: 2.520 to 102.681, *p* = 0.04), Δright ovarian volume (*β* = 1.881, 95% CI: 0.062 to 3.699, *p* = 0.04), and Δleft ovarian volume (*β* = 0.971, 95% CI: 0.019 to 1.923, *p* = 0.05).

**Table 7:** Multiple lineal regression results for DGTP intake associated with the change of obesity-related parameters after intervention among obese girls^1^.

	Independent variable	Obesity- or sexual development-related parameter	*β*	Standardized *β*	*t*	95% CI	*p*
Model 1^2^	Groups (1 DGTP group, 2 Control group)	ΔBMI, kg/m^2^	−0.048	−0.018	−0.139	(−0.743, 0.647)	0.89
		ΔPBF, %	1.712	0.183	1.419	(−0.702, 4.127)	0.16
		ΔUA, μmol/L	48.747	0.306	2.347	(7.100, 90.394)	0.02^†^
		ΔUterine volume, cm^3^	0.074	0.019	0.142	(−0.974, 1.122)	0.89
		ΔRight ovarian volume, cm^3^	0.705	0.161	1.261	(−0.414, 1.824)	0.21
		ΔLeft ovarian volume, cm^3^	0.726	0.232	1.809	(−0.078,1.530)	0.08
		ΔE2, pmol/L	−2.324	−0.024	−0.185	(−27.445, 22.798)	0.86
		ΔFSH, IU/L	−0.558	−0.194	−1.505	(−1.300, 0.184)	0.14
		ΔLH, IU/L	−0.238	−0.185	−1.470	(−0.563, 0.086)	0.15
Model 2^3^	Groups (1 DGTP group, 2 Control group)	ΔBMI, kg/m^2^	−0.265	−0.128	−0.402	(−1.733, 1.203)	0.70
		ΔPBF, %	2.810	0.329	1.066	(−3.063, 8.683)	0.31
		ΔUA, μmol/L	55.172	0.347	2.293	(6.328, 104.015)	0.03^‡^
		ΔUterine volume, cm^3^	0.301	0.132	0.943	(−0.347, 0.949)	0.35
		ΔRight ovarian volume, cm^3^	1.935	0.513	2.58	(0.336, 3.533)	0.02^‡^
		ΔLeft ovarian volume, cm^3^	989.249	0.313	2.004	(−6.136, 1984.635)	0.05
		ΔE2, pmol/L	−27.489	−0.281	−0.740	(−111.473, 56.495)	0.48
		ΔFSH, IU/L	−1.476	−0.489	−1.560	(−3.584, 0.632)	0.15
		ΔLH, IU/L	0.046	0.060	0.141	(−0.687, 0.780)	0.89
Model 3^4^	Groups (1 DGTP group, 2 Control group)	ΔBMI, kg/m^2^	−0.973	−0.469	−2.172	(−2.125, 0.179)	0.08
		ΔPBF, %	2.932	0.303	2.190	(0.214, 5.650)	0.04^¶^
		ΔUA, μmol/L	52.601	0.331	2.137	(2.520, 102.681)	0.04^¶^
		ΔUterine volume, cm^3^	0.285	0.125	0.805	(−0.438, 1.008)	0.43
		ΔRight ovarian volume, cm^3^	1.881	0.498	2.253	(0.062, 3.699)	0.04^¶^
		ΔLeft ovarian volume, cm^3^	0.971	0.307	2.060	(0.019, 1.923)	0.05^¶^
		ΔE2, pmol/L	−19.181	−0.196	−0.355	(−151.283, 112.921)	0.74
		ΔFSH, IU/L	−1.682	−0.557	−1.166	(−5.210, 1.847)	0.29
		ΔLH, IU/L	−0.199	−0.257	−0.448	(−1.288, 0.889)	0.67

^1^BMI, body mass index; DGTP, decaffeinated green tea polyphenols; E2, estradiol; FSH, follicle-stimulating hormone; LH, luteinizing hormone; PBF, percentage of body fat; UA, serum uric acid.

^2^Model 1: regression with age controlled, ^†^p < 0.05.

^3^Model 2: regression with parents’ education, family income, weekly physical activity, sleep time and past eating habits controlled building upon model 1, ^‡^p < 0.05.

^4^Model 3: regression with energy, protein, lipids and carbohydrate intakes at baseline and after intervention controlled building upon model 2, ^¶^p < 0.05.

No participants withdrew from the study because of discomfort or adverse events related to the intervention. No adverse reactions and discomfort indigestion were noted and reported in the study period. The changes in their serum trace elements, hepatic and renal function and complete blood count showed no significant differences between the two groups and remained within the normal ranges (not shown in tables). Some participants reported that the size of the capsule was large, which was a little difficult to swallow for them.

## Discussion

This study demonstrated that short-term DGTP daily consumption effectively ameliorated obesity and reduced the risk of hyperuricemia among girls with obesity. DGTP might also be effective in preventing early sexual development in girls with obesity. No adverse effects were reported. To our knowledge, this is the first randomized controlled trial study examining the effect of DGTP on obesity and precocious puberty in children, and these findings provided valuable evidence for DGTP clinical application.

In our study, after the intervention, the BMI, WC, WHR, FFM, and PBF in the DGTP group decreased significantly, and DGTP could also reduce ΔPBF after controlling confounders. These findings suggested that DGTP could contribute to a decrease in obesity in children, which were similar with the results of some previous studies (22, 31, 33–35) on GTP for adult weight reduction and its reduction effects on BMI, WHR, and PBF. The mechanisms of GTP in reducing body weight and fat were discussed, such as GTP could inhibit the activities of gastrointestinal enzymes involved in lipids and sugar absorption and metabolism (36–38), or EGCG had an effect on activating like AMP-activated protein kinase, to effectively promote the decomposition and inhibit the formation of body fat (39, 40).

Also, this study reported that DGTP could reduce the concentration of uric acid in children, and in the adjusted model, the significant effect still existed. Some *in vitro* and *in vivo* studies (41, 42) showed that GTP might have the effect of lowering uric acid through decreasing uric acid production and increasing uric acid excretion *via* the inhibition of xanthine oxidase activity and glucose transporter-9 expression and the promotion of organic anion transporter-1 expression.

In the control group, the BMI, WC, and WHR of participants had significant reduction after intervention as well. This change might be due to the professional instruction on diet and physical activities by doctors and dietitians in the Clinical Nutrition Department. With those instructions, participants had developed healthier dietary patterns and habits and had improved health awareness.

In the current study, after DGTP intervention, the ovarian growth of girls with obesity appeared as a “REVERSAL” phenomenon, which is reduced ovarian volumes and reduced number of follicles. This phenomenon might confirm the positive effect of DGTP on postponing early sexual development among girls with obesity. The participants we recruited were simple obese girls and they have not yet entered puberty. Since their hypothalamic–pituitary–gonadal axis has not been officially launched, after the effective intervention, the delayed ovarian development seemed reasonable. In addition, the sex hormone concentrations increased in both groups after 12 weeks significantly but slightly. Overall, they remained at low concentrations, far below the hormone concentration standards of puberty for girls, which also indicated that these girls with obesity have not entered the sexual developmental stage. Certainly, further follow-up is needed to determine whether the final onset of menarche is delayed among girls with obesity. Also, the mechanisms of DGTP to prevent early sexual development in girls with obesity need to be further explored. Some previous studies suggested that leptin was considered likely to be the important signal between obesity and puberty (27, 43, 44), and GTP has been found to suppress the effects of leptin or inhibit the expression of leptin in adipose tissue in obese rats and adults (45, 46). GTP might reduce the expression of leptin in adipose tissue, thereby inhibiting puberty by regulating gonadotrophin-releasing hormone secretion and postponing the initiation of the hypothalamic–pituitary–gonadal axis.

These findings are of great significance in preventing precocious puberty and ameliorating obesity and high uric acid or hyperuricemia among girls with obesity. Green tea polyphenols, as widely accepted natural functional food ingredients, have been further explored and used in a new field of clinical nutrition and pediatric endocrinology. The results of this study also showed great economic value and market of GTP.

However, some limitations also existed in the current study. First, the dropout rate was 26.2% in this study, which was a little high in a RCT. As we described before, 9 of the 22 dropouts could not pay study visits on time due to the COVID-19 pandemic in early 2020 in China. The suddenly new outbreak of COVID-19 hit all the medical resources nationally and people were too scared and observed social distancing strictly, let alone went out to go to the hospital. Our study was unavoidably affected by the situation that the nine participants were way beyond their 12-week study visit when the pandemic alleviated after April 2020; thus, we did not take their post-treatment data into analysis due to the obvious bias. Referring to the study process before the pandemic, we suppose the dropout rate should be much lower without the impact of COVID-19. Second, as this is the first study to identify the effect of DGTP on precocious puberty in children, we have no reference to calculate our sample size. Therefore, referring to the only previous RCT about green tea catechins on children in Japan (22), the sample size of participants was 40 (catechin group; *n* = 21, control group; *n* = 19), and we considered our sample size would also be meaningful and, most importantly, provide effective evidence in this field. We also consulted some experts in epidemiology, pediatric endocrinology, and clinical nutrition about our study design and sample size, and they all agreed that this study could provide some preliminary implications if no previous literature helps to calculate the sample size, but small sample size was still one of the limitations. Third, human error may occur during testing and data collection. Participants all started at different times. The testing was done by different clinicians and thus the interpretation of results might be affected by their skills and experiences, e.g., the B-scan ultrasound imaging reports of uterus, ovaries, and breast tissues. Fourth, other parameters, such as sex hormone-binding globulin, could help to further explain the mechanisms between obesity and precocious puberty, but have not been measured in the present study. Finally, the 12-week 400 mg/day DGTP intervention period might not be sufficient to determine the final pubertal change of girls with obesity. This study is a preliminary explorer in this vacant field, and longer follow-up is also needed to determine whether their final age of menarche onset was delayed and the adult heights were as excepted.

## Conclusions

This study demonstrated that short-term DGTP daily consumption effectively ameliorated obesity and reduced the risk of hyperuricemia among girls with obesity. DGTP might also be effective in preventing early sexual development in girls with obesity. No adverse effects were reported. These findings provided valuable evidence for the clinical application of DGTP in children.

## Data Availability Statement

The original contributions presented in the study are included in the article/supplementary material. Further inquiries can be directed to the corresponding author.

## Ethics Statement

The studies involving human participants were reviewed and approved by the Ethics Committee of Xinhua Hospital (Shanghai, China). Written informed consent to participate in this study was provided by the participants’ legal guardian/next of kin.

## Author Contributions

LX and XS conceptualized and designed the research, conducted the intervention to this study’s setting, contributed the data collection instruments, collected data, performed statistical analysis, wrote the paper, revised the manuscript, and had primary responsibility for final content. QT conceptualized and designed the study, coordinated and supervised data collection, supported training of data collection staff, and critically reviewed the manuscript for important intellectual content. DY, QG, HZ, and XW supported intervention to the setting and collected data. ZY helped with the initial manuscript and critically reviewed and revised the manuscript. All authors contributed to the article and approved the submitted version.

## Funding

This work is supported by the National Natural Science Foundation of China (No. 81773407).

## Conflict of Interest

The authors declare that the research was conducted in the absence of any commercial or financial relationships that could be construed as a potential conflict of interest.

## Publisher’s Note

All claims expressed in this article are solely those of the authors and do not necessarily represent those of their affiliated organizations, or those of the publisher, the editors and the reviewers. Any product that may be evaluated in this article, or claim that may be made by its manufacturer, is not guaranteed or endorsed by the publisher.
